# Premetastatic niche and tumor-related leukocytosis: a close relationship that cannot be ignored in uterine cancer patients

**DOI:** 10.18632/oncotarget.26425

**Published:** 2018-12-11

**Authors:** Seiji Mabuchi, Tomoyuki Sasano, Naoko Komura

**Affiliations:** Seiji Mabuchi: Department of Obstetrics and Gynecology, Osaka University Graduate School of Medicine, Osaka, Japan

**Keywords:** leukocytosis, MDSC, G-CSF, uterine cancer, premetastatic niche

Despite recent advances in cancer screening; new radiotherapy techniques; and chemotherapies, including targeted agents, metastasis is the primary cause of death in cancer patients [1].

Systemic inflammatory responses have gained attention as a prognostic indicator in cancer patients, and an increasing number of reports focusing on leukocytosis, neutrophilia, thrombocytosis, the neutrophil-lymphocyte ratio, or the platelet-lymphocyte ratio have been published in the past 10 years [2, 3]. Tumor-related leukocytosis (TRL) is a paraneoplastic syndrome, which is occasionally (10-15%) encountered in cancer patients, and it is associated with a poor prognosis [4, 5]. In studies of uterine cervical cancer, we have previously demonstrated that the granulocyte-colony stimulating factor (G-CSF) produced by tumor cells causes TRL and that myeloid-derived suppressor cells (MDSC) are responsible for the rapidly growing and radio/chemo-resistant natures of TRL-positive cancer [4, 5].

MDSC, which are immature myeloid cells that exhibit immunosuppressive activity, are key components of the tumor microenvironment. Accumulating evidence suggests that MDSC promote cancer progression by suppressing anti-tumor immune responses and stimulating tumor cell proliferation, epithelial-mesenchymal transition, cancer stem cell formation, and angiogenesis [6]. Thus, MDSC are now regarded as an important therapeutic target for cancer treatment as well as a predictive biomarker of treatment outcomes.

Although previous case series have highlighted the aggressive nature of TRL-positive cancer [7], the metastatic potential of TRL-positive cancer as well as the role of MDSC in the metastatic processes of gynecological cancers remain to be elucidated.

**Figure 1:**
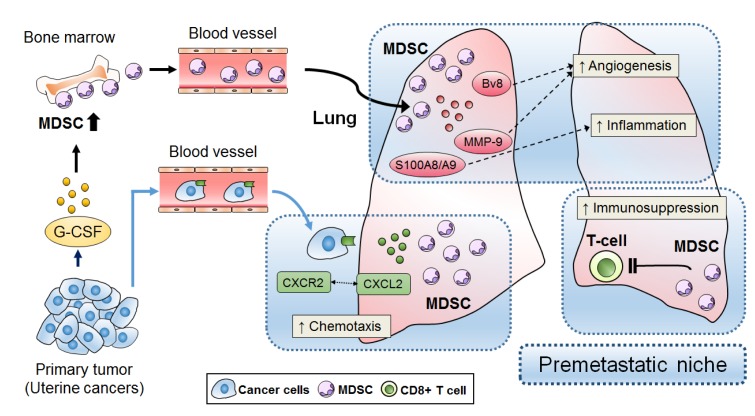
Proposed premetastatic niche mechanism in TRL-positive uterine cancer MDSC that develop in response to tumor-derived G-CSF contribute to premetastatic niche formation.

Recently, we demonstrated, for the first time, that uterine cervical and endometrial cancers that display TRL or neutrophilia represent a distinct clinical entity with a highly metastatic nature [8]. Preclinical findings obtained in mouse models of cervical cancer and studies involving patient-derived samples have suggested that this highly metastatic nature might be the result of tumor-derived G-CSF, G-CSF-induced MDSC, and MDSC-mediated premetastatic niche formation. In the current study, to mimic the clinical setting found in cases of TRL-positive cancer, we employed a mouse model bearing G-CSF expressing cervical cancer cells and found that the MDSC in premetastatic lungs secreted Bv8, matrix metalloproteinase-9, and S100A8/A9. Moreover, we demonstrated that the MDSC in premetastatic lungs secreted the proinflammatory chemokine, chemokine (C-X-C motif) ligand 2 (CXCL2), which attracted C-X-C motif chemokine receptor 2 (CXCR2)-expressing cancer cells. This indicated that the MDSC induced by tumor-derived G-CSF contribute to the metastatic process in TRL-positive cancer by facilitating the formation of a premetastatic niche; i.e., a proinflammatory, immunosuppressive, and proangiogenic environment (Figure 1). Importantly, MDSC inhibition attenuated premetastatic niche formation and prevented cancer cell metastasis. Regarding the limitations of the present study, we are concerned that the use of nude mice might have affected our results. A recent study suggested that lung metastasis is more frequently observed in immunodeficient mice than in immunocompetent mice [9]; therefore, we are planning to perform verification experiments using immunocompetent mice.

Our preclinical and clinical findings have several important clinical implications. First, by performing simple and low-cost peripheral blood examinations, it might be possible to identify patients who are at greater risk of developing metastasis, which would allow careful pretreatment work-ups or post-treatment follow-up examinations to be conducted in such cases. Second, TRL has been observed not only in uterine cervical or endometrial cancers, but also in other gynecological cancers and non-gynecological malignancies. Although ours is the only study to investigate the association between TRL and MDSC in cancer patients [8], increased numbers of circulating MDSC or tumor-infiltrating MDSC have been detected in patients with various types of cancer, and MDSC were shown to be associated with an advanced clinical stage or shorter survival. Thus, we consider that strategies that target MDSC or MDSC-mediated premetastatic niche formation might hold the key to improving the prognosis of cancer patients that display TRL.

Currently, no specific inhibitors of human MDSC are available. Given the complicated network of mediators involved in the expansion, recruitment, and activity of MDSC, a variety of different therapeutic strategies have been developed to target them. Until MDSC-specific inhibitors are available, the use of indirect MDSC-inhibiting methods that target these mediators might be a reasonable approach. The results of our investigation suggest that targeting the CXCL2/CXCR2 axis could be a promising treatment strategy for TRL-positive cancer [8]. Other possibilities include the use of all-trans-retinoic acid, vitamin D, phosphodiesterase 5 inhibitors, gemcitabine, lurbinectedin, or 5-FU, as these molecules have been shown to reduce the number of circulating MDSC in preclinical or clinical settings [6, 10]. We hope that the ability of these existing agents to inhibit MDSC will be evaluated in clinical trials in future.

Our findings raise several questions: (1) Does MDSC-mediated premetastatic niche formation contribute to the metastatic process in other TRL-positive cancers? (2) Do other tumor-derived mediators contribute to the induction of TRL? (3) What is the mechanism by which G-CSF expression is upregulated in TRL-positive cancer? (4) Do other tumor-derived mediators or chemokine-chemokine receptor axes have effects in TRL-positive cancer patients? Consequently, further investigations will be required to elucidate the nature of TRL-positive cancer.

As suspected in earlier case series, TRL was demonstrated to be associated with a significantly increased risk of visceral organ metastasis in patients with uterine cervical or endometrial cancer. Our study provides valuable preclinical and clinical information, which might aid the development of novel MDSC-targeting treatments or chemoprevention strategies for this patient population. The utility of pretreatment leukocyte count for predicting cancer metastasis, the significance of MDSC in the metastatic process, as well as the efficacy of MDSC-targeting therapies in TRL-positive patients should be investigated further in clinical trials, especially in prospective studies.
